# The diagnostic performance of ^18^F-DCFPyL PET in patients with suspected prostate cancer: A systemic review and meta-analysis

**DOI:** 10.3389/fonc.2023.1145759

**Published:** 2023-03-07

**Authors:** Wenyang Pang, Shulin Cheng, Zhongbo Du, Shuang Du

**Affiliations:** ^1^ Department of Urology, Affiliated Hospital of North Sichuan Medical College, Nanchong, Sichuan, China; ^2^ Department of Dermatology, Affiliated Hospital of North Sichuan Medical College, Nanchong, Sichuan, China

**Keywords:** ^18^F-DCFPyL PSMA PET, prostate cancer, diagnostic performance, meta-analysis, systemic review and meta-analysis

## Abstract

**Introduction:**

Our meta-analysis aimed to evaluate the diagnostic value of ^18^F-DCFPyL prostate-specific membrane antigen (PSMA) PET in patients with suspected prostate cancer.

**Methods:**

We searched for articles that evaluate the diagnostic value of ^18^F-DCFPyL PSMA PET in patients with suspected prostate cancer in PubMed, Embase, Cochrane Library, and Web of Science until 1 August 2022. Using the QUADAS-2 instrument, two researchers independently assessed the effectiveness of the studies that were included. The four-grid table data were analyzed by Meta-disc1.4 and Stata 16.0 software. The heterogeneity of each study was tested.

**Results:**

A total of five studies with 258 patients were included, and the pooled sensitivity and specificity of ^18^F-DCFPyL PSMA PET for primary prostate cancer were 0.92 (95% confidence interval (CI): 0.85–0.96) and 0.59 (95% CI: 0.08–0.96), respectively. ^18^F-DCFPyL PSMA PET was successful in detecting primary prostate cancer, with an area under the curve (AUC) of 0.92 (95% CI: 0.89–0.94).

**Conclusions:**

^18^F-DCFPyL PSMA PET has a strong predictive value for primary prostate cancer and is an effective method for the non-invasive diagnosis of prostate cancer. More prospective articles were needed.

## 1 Introduction

Prostate cancer (PCa) is one of the major health problems plaguing elderly men. According to statistical analysis, there were 1.4 million new cases of prostate cancer patients worldwide in 2020 (1). Accurate early diagnosis is essential and necessary in individuals with PCa to provide the best care and enhance prognosis (2).

At present, the diagnostic methods of PCa include digital rectal examination (DRE), prostate-specific antigen (PSA), and transrectal ultrasound (TRUS). The gold standard is still the pathological result of puncture biopsy, but there is a risk of bleeding, severe infection, urinary retention, and so on. At present, it is a question that we need to consider whether there are other ways to reduce unnecessary prostate biopsy or even replace puncture biopsy to diagnose prostate cancer to avoid the above risks (3, 4).

Prostate-specific membrane antigen (PSMA) is a type II glycoprotein with a wide range of extracellular domains (44-750A). The protein is rarely expressed in normal prostate tissues but is highly upregulated and overexpressed in PCa cells and tumor vascular cells (5–7). With rapid development, PSMA is playing a transformative role in diagnosis and treatment (8). PET/CT imaging technology targeting ^68^Ga-PSMA has developed rapidly in recent years. The generator, however, produced the nuclide ^68^Ga, which had a short half-life and significant electron energy, which reduced its clinical application. In clinical practice, the most commonly used positron nuclide was ^18^F-DCFPYL. It is based on a glutamate-urea-lysine structure. When compared with ^68^Ga-PSMA-11, some of its traits include strong affinity, favorable *in vivo* pharmacokinetics, and good solubility and may have a higher detection rate for small lesions. Therefore, the performance is better, and it is easier to be widely used in clinical practice (9–11).

At present, the research that evaluates ^18^F-DCFPyL PSMA PET in the diagnosis of suspect PCa is small in scale. Therefore, we collected relevant pieces of literature and summarized their data for meta-analysis to more accurately evaluate the diagnostic value of ^18^F-DCFPyL PSMA PET in patients with suspected prostate cancer.

## 2 Methods

### 2.1 Search strategy

We searched relevant published literature by computer, and the search databases included PubMed, Embase, Cochrane Library, and Web of Science. The search time was from the establishment of the database to 1 August 2022. The search terms are the following phrases: DCFPyL, Prostatic Cancer, Prostatic Cancer, Prostate Cancer, Prostate Cancer, Prostatic Neoplasm, Prostate Neoplasm, Prostate Neoplasms, Prostate tumor, and prostatic tumor. Two researchers independently combined computer search with manual search to avoid missing relevant literature.

### 2.2 Inclusion criteria

Articles that met the following requirements were only considered for inclusion: 1) untreated patients with suspected prostate cancer, who are patients with prostate abnormalities found in DRE or TRUS, MRI examination, or PSA screening abnormalities. 2) ^18^F-DCFPyL PSMA PET scan was performed. 3) The number of subjects ≥10. 4) The reference standard for prostate cancer was a histopathological biopsy.

### 2.3 Exclusion criteria

The following cases are excluded: 1) patients with biochemical recurrence; 2) patients with metastases; 3) case reports, abstracts, comments, letters, reviews, or meta-analyses; 4) data to construct a four-grid table cannot be extracted.

### 2.4 Quality assessment

Using the QUADAS-2 instrument, two researchers independently assessed the effectiveness of the studies that were included. The patient selection, index test, reference standard, and flow and timing were the domains that were utilized to assess each study. The bias risk determined whether these domains’ applicability was rated as “high”, “poor”, or “unclear”. The disputes among the researchers were ultimately settled by agreement.

### 2.5 Data extraction

A total of two researchers independently extracted data for each study. General information, characteristics of the literature, patient characteristics, technical information, and results for the total number of patients, True positive (TP), False positive (FP), True negative (TN) and False negative (FN)—all of which are counted—were all extracted from the data. If these values were not available, calculations were made using the findings of sensitivity, specificity, positive predictive value (PPV), and negative predictive value (NPV) tests.

### 2.6 Statistical analysis

The four-grid table data were analyzed by Meta-disc1.4 and Stata 16.0 software. Spearman’s rank correlation coefficient was used to evaluate threshold effect performance, and a *p*-value <0.05 indicates that the threshold effect is observed (12). If a threshold effect is not observed, the Stata software package midas command was used to analyze the four-grid data. With the use of a bivariate random-effects model, the pooled sensitivity and specificity for ^18^F-DCFPyL PSMA PET were reported as estimates with 95% confidence intervals (CIs). The summary receiver operating characteristic curve and area under the curve (AUC) were generated by using the summary receiver operating characteristic (SROC) model (13). The heterogeneity of each study was evaluated by heterogeneity index I^2^ and χ^2^ test (14). I^2^ quantifies heterogeneity by calculating the proportion of variation due to heterogeneity rather than due to chance. Homogeneity among the studies was considered to be low, moderate, or high when the I^2^ value was 25%, 50%, or 75%, respectively. The sources of heterogeneity were identified with a meta-regression analysis when there was obvious heterogeneity. Stata 16.0 was used to make a Deek’s funnel plot to evaluate publication bias (15).

## 3 Results

### 3.1 Literature search

**Figure 1:**
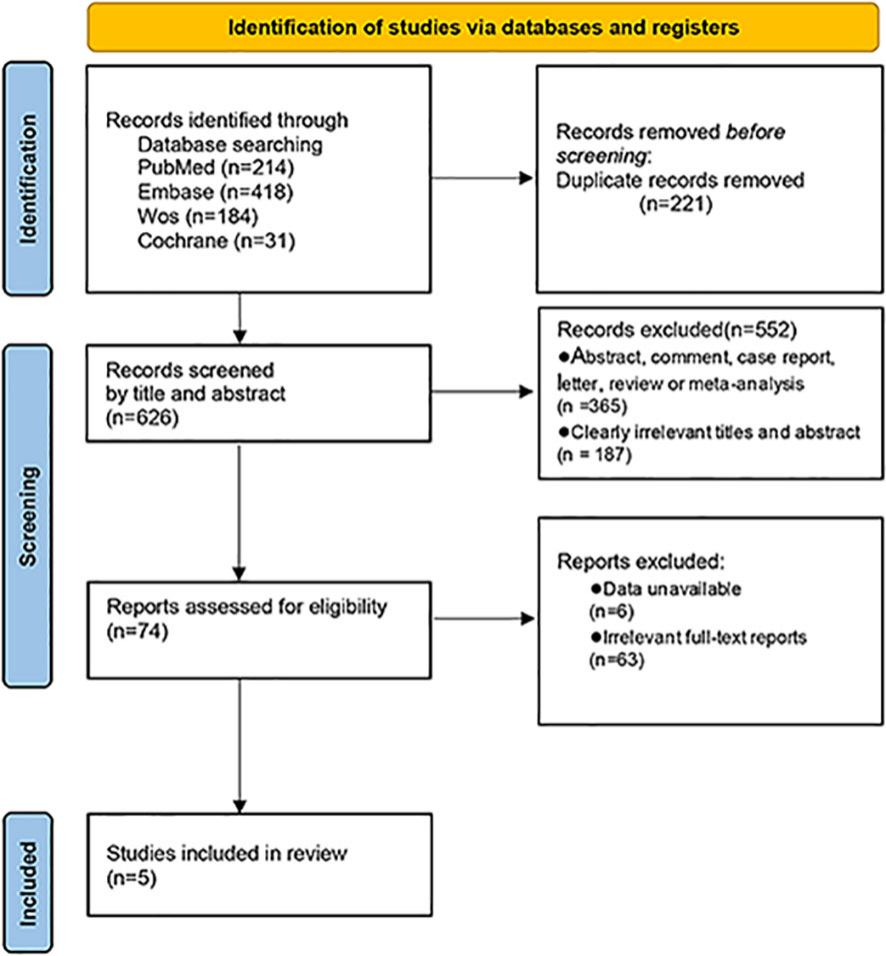
Flow diagram for study selection.

Through the search of PubMed, Cochrane Library, Web of Science databases, and Embase, 847 related articles were initially detected. Endnote X9 software was used for literature management, and 221 duplicate references were deleted. After reading the title and abstract, 552 irrelevant articles were excluded. After reading the full text of the remaining 74 articles that may meet the requirements, 63 articles met the exclusion criteria after reading the full text, and six articles could not directly or indirectly extract the four-grid table data. Finally, five papers were selected (16–20) (**Figure 1**).

### 3.2 Study evaluation

**Figure 2:**
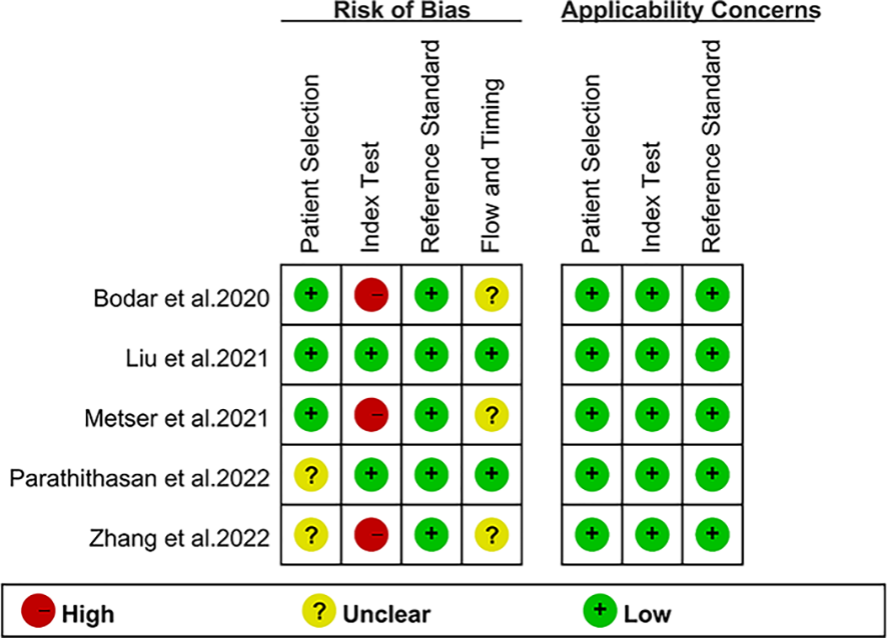
Study risk of bias and applicability concerns summarized.

The investigations and patient factors of the five papers, which covered 258 patients, are summarized in **Table 1**, and the parameters and ^18^F-DCFPyL PSMA PET reference standards are listed in **Table 2**. Two of the five studies were designed to prospectively address this study question (16, 17). In four studies, positive PSMA PET results were evaluated per patient, whereas in one study, the data were evaluated per lesion (16). **Figure 2** displays the overall findings for each study’s bias risk and applicability issues. The standard of the accepted consensus inclusion research determines the final result.

**Table 1:** Study and patient characteristics of the included studies.

Author	Year	Study characteristics			Patient characteristics			
		Country	Study design	Analysis	No. of patients	Mean age ± SD	PSA level (ng/ml)	Gleason score
Parathithasan et al. (20)	2022	Australia	Retro	PB	65	67 (44–80)	Mean ± SD: 14.3 ± 11.6	Gleason score ≤ 6 (6.2%) Gleason score = 7 (58.5%) Gleason score ≥ 8 (35.3%)
Bodar et al. (16)	2020	Netherlands	Pro	LB	30	68.5	Median: 11.1	Gleason score ≤ 6 (0%) Gleason score = 7 (53.3%) Gleason score ≥ 8 (46.7%)
Liu et al. (19)	2021	China	Retro	PB	52	65 (48–79)	Mean ± SD: 18.3 ± 16.0	Gleason score ≤ 6 (9.6%) Gleason score = 7 (28.8%) Gleason score ≥ 8 (61.6%)
Metser et al. (17)	2021	Canada	Pro	PB	55	65.1 ± 7.2	Mean ± SD: 8.8 ± 5.3	NA
Zhang et al. (18)	2022	China	Retro	PB	56	68 (43–83)	Median (range): 20.4 (1.9–1000)	Gleason score ≤ 6 (1.8%) Gleason score = 7 (32.1%) Gleason score ≥ 8 (55.3%) Unknown (10.7%)

PB, patient-based; LB, lesion-based; Pro, prospective; Retro, retrospective; PSA, prostate-specific antigen. NA, Not Applicable.

**Table 2:** Technical aspects of included studies.

Author	Year	Scanner modality	Ligand dose	Image analysis	Total	TP	FP	FN	TN
Metser et al. (17)	2021	Biograph mMR, Siemens Healthcare, Erlangen, Germany, PET/MRI	329.5 MBq/kg	Quantitative	55	39	12	3	1
Zhang et al. (18)	2022	Siemens Medical Solutions, Knoxville, TN, PET/CT	4.44 MBq/kg	Quantitative	56	45	0	5	6
Bodar et al. (16)	2020	Philips Healthcare^®^, NL/USA—PET/CT system	313 MBq/kg	Quantitative	420	103	9	19	289
Liu et al. (19)	2021	Biograph 64, Siemens Healthcare, PET/CT Biograph mMR; Siemens Healthcare, PET/MRI	NA	Quantitative	52	40	2	3	7
Parathithasan et al. (20)	2022	General Electric Medical Systems, Milwaukee WI, PET/CT	250 MBq/kg	Quantitative	65	59	4	2	0

### 3.3 Diagnostic performance

**Figure 3:**
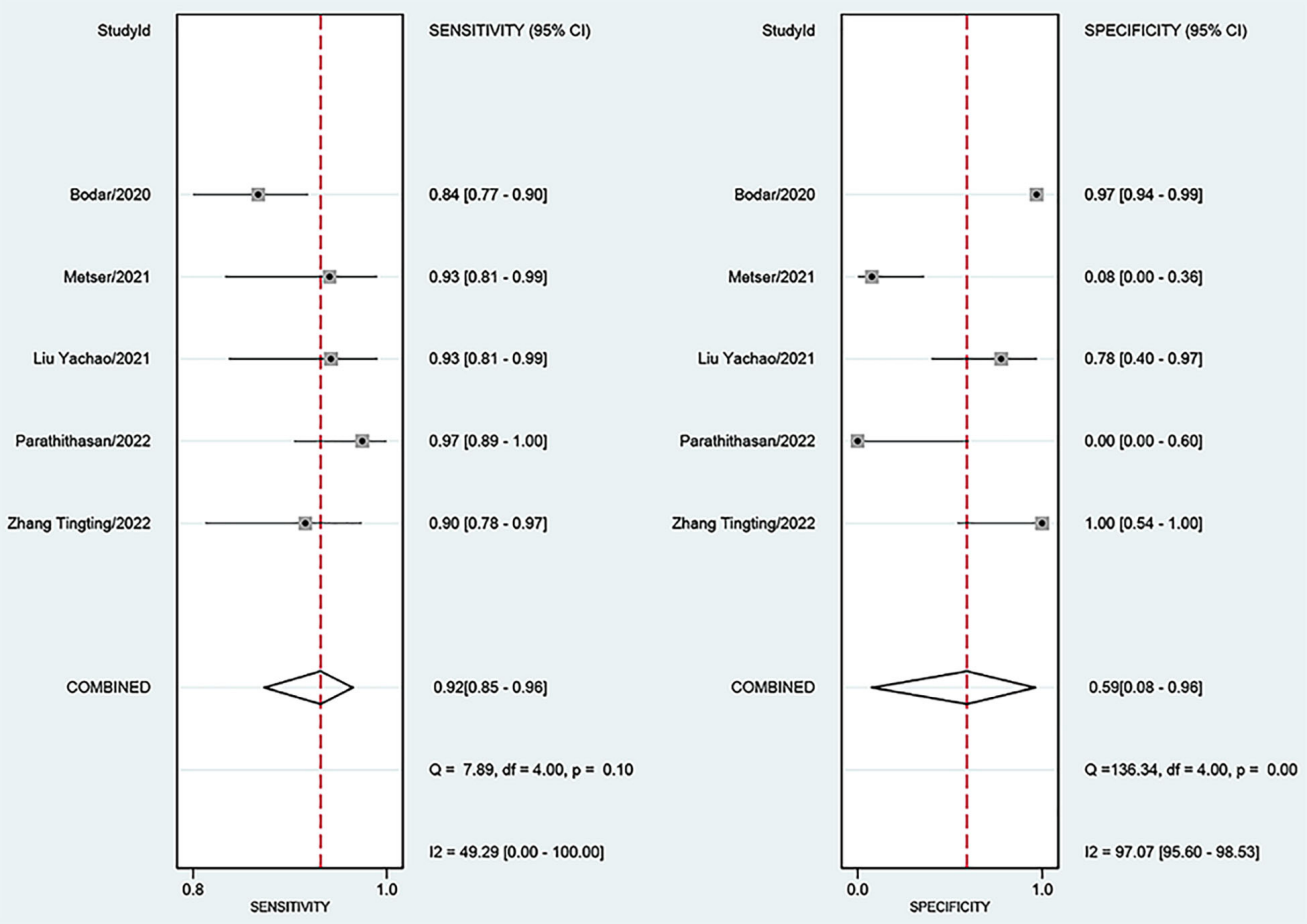
Forest plot showing the sensitivity and specificity of ^18^F-DCFPyL PSMA PET for primary prostate cancer. PSMA, prostate-specific membrane antigen.

**Figure 4:**
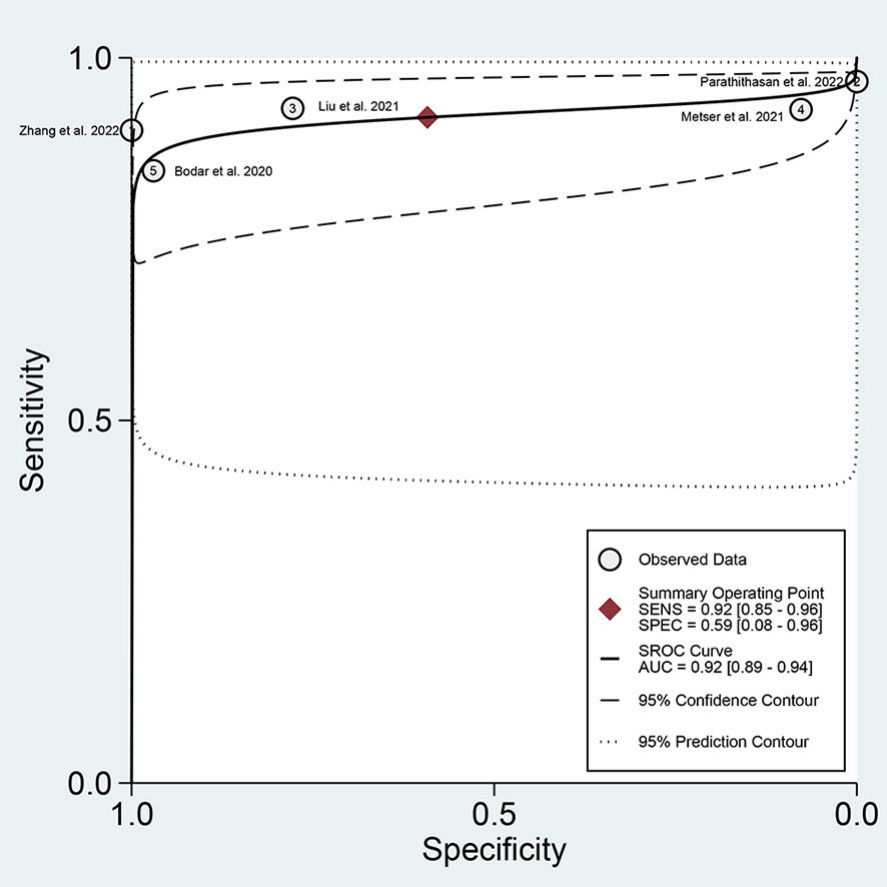
SROC curve for ^18^F-DCFPyL PSMA PET with an AUC value. SROC, summary receiver operating characteristic; PSMA, prostate-specific membrane antigen; AUC, area under the curve.

**Figure 5:**
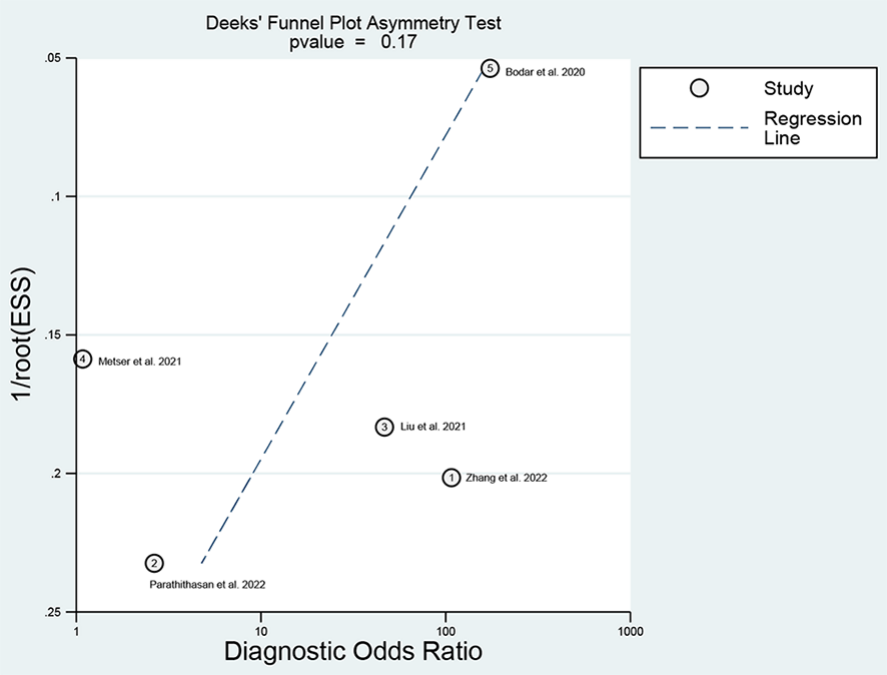
Deeks’ test.

**Figure 6:**
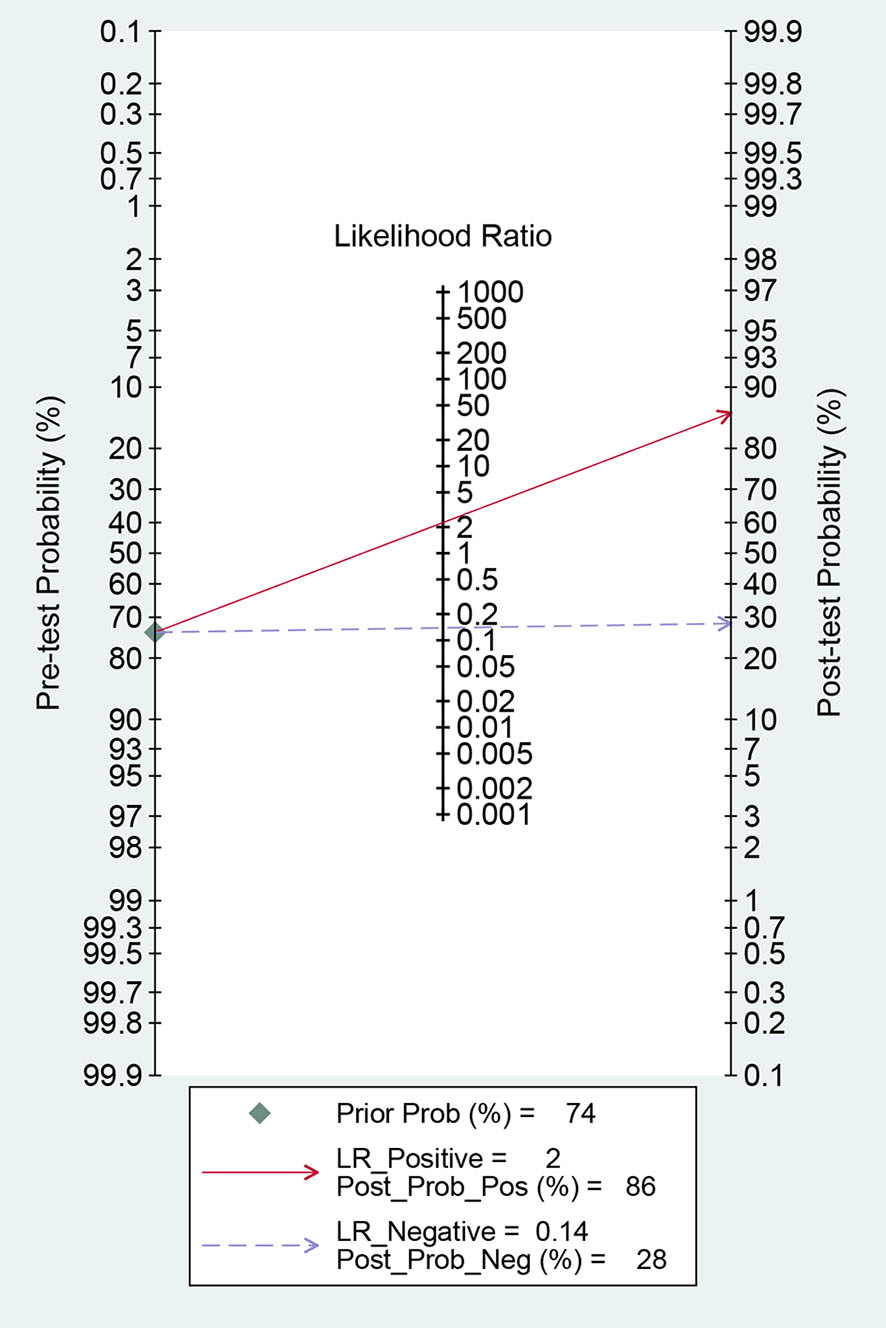
The Fagan plot; the pre-test probability is the mean of prevalence in the five articles.

For ^18^F-DCFPyL PSMA PET, the results of Spearman’s rank correlation coefficient demonstrated that a threshold effect was not observed (Spearman’s correlation coefficient = 0.700, *p* = 0.188). The pooled sensitivity and specificity of ^18^F-DCFPyL PSMA PET for primary prostate cancer were 0.92 (95% CI: 0.85–0.96) and 0.59 (95% CI: 0.08–0.96), respectively (**Figure 3**). **Figure 4** shows the SROC curve for the AUC of 0.92 of ^18^F-DCFPyL PSMA PET (95% CI: 0.89–0.94). We tried to find out the cause of heterogeneity by using meta-regression analysis. It demonstrated that diverse races (specificity *p* < 0.001) and analysis (specificity *p* < 0.001) were two potential causes of heterogeneity (**Table 3**). No publication bias was discovered (*p* = 0.17), as shown in **Figure 5**. To facilitate clinical analysis, the Fagan diagram was created. It might be seen in **Figure 6**. The ^18^F-DCFPyL PSMA PET posttest probability was 86%, which was greater than the pretest likelihood of 74%.

**Table 3:** Meta-regression analysis for ^18^F-DCFPyL PET in detecting suspected prostate cancer.

Covariate	Studies, n	Sensitivity (95% CI)	*p*-Value	Specificity (95% CI)	*p*-Value
Analysis			<0.001		<0.001
Patient-based	4	0.94 (0.90–0.97)		0.42 (−0.28 to 1.00)	
Lesion-based	1	0.84 (0.75–0.94)		0.97 (0.83 to 1.00)	
Race			0.19		<0.001
White	3	0.92 (0.84–0.99)		0.24 (−0.48 to 0.96)	
Others	2	0.92 (0.87–0.98)		0.95 (0.75 to 1.00)	
Study design			0.02		0.80
Retrospective	3	0.94 (0.89–0.99)		0.67 (−0.50 to 1.00)	
Prospective	2	0.89 (0.81–0.97)		0.61 (−0.29 to 1.00)	

## 4 Discussion

PCa has become one of the major malignant tumors in the male reproductive system that causes more trouble to older men, which occurs in the prostate epithelium. The diagnosis of tumors has received more attention in recent years in cancer research investigations, which include but are not limited to blood biomarkers, tissue pathology analysis, and imaging methods (21). It has become clear that the best cancer screening techniques should be precise, non-invasive, and practical, especially at the early stage (22). However, the biological behavior of PCa and its clinical manifestations have a great deal of variation, which makes the diagnosis extremely challenging. Therefore, high sensitivity and precision imaging equipment are essential because most patients miss the ideal therapy window.

PSMA is a transmembrane protein located in prostate epithelial cells. Compared with normal prostate tissue, almost all prostate cancer cells can highly express PSMA in the cell membrane. Studies related to ^68^Ga-PSMA PET have shown good diagnostic and staging values in primary prostate cancer (23–25). However, its clinical application is limited because of the short half-life and low PET images. However, the new imaging agent ^18^F-DCFPYL is popular because of its high affinity and good *in vivo* pharmacokinetics. The clinical comparison showed that its various properties were better than those of ^68^GA-PSMA-11 (10, 11, 26).

Therefore, in this study, we evaluated the diagnostic value of ^18^F-DCFPyL PSMA PET in patients with suspected prostate cancer. In a previous meta-analysis, the combined sensitivity estimates for ^18^F-DCFPyL PSMA PET in identifying prostate cancer was 0.91, and the specificity was 0.90. However, it was restricted to research comparing PET/CT to CT, but our literature was not. In contrast, for analysis, patients with biochemical recurrence or distant metastasis were mainly included, whereas our study involved untreated patients with suspected prostate cancer. The Bodar et al. (16) prospective studies showed that the sensitivity of ^18^F-DCFPyL PSMA PET for prostate cancer diagnosis was 61.4%, and the specificity was 88.3%. Metser et al. (17) also conducted a prospective study and concluded a sensitivity of 86% and a specificity of 32%. In our study, the sensitivity and specificity of ^18^F-DCFPyL PSMA PET for the diagnosis of prostate cancer are 0.92 (95% CI: 0.85–0.96) and 0.59 (95% CI: 0.08–0.96), respectively. ^18^F-DCFPyL PSMA PET showed better accuracy than multiparameter MRI and CT in the diagnosis of prostate cancer. Meissner et al. (27) described for the first time a possible biopsy-free diagnostic pathway for PC in selected men with a high suspicion of significant malignancy in both multiparametric MRI (mpMRI) and PSMA PET. We expect that in the near future, under certain circumstances, patients will be exempted from biopsies.

There were still certain limitations to this study. First, the number of articles included in the study is limited, and the total number of patients is not large enough, which may bring selection bias. Second, there are not enough prospective studies in the included articles, which may affect the sensitivity and specificity. In the end, when we combined the general PET specificity, there was greater heterogeneity. The cause of heterogeneity was examined using meta-regression analysis. It demonstrated that diverse races (specificity *p <*0.001) and the patient-based and lesion-based analyses (specificity *p <*0.001) were two potential causes of heterogeneity. Of course, there could be other causes that are sources of heterogeneity.

## 5 Conclusions

In conclusion, ^18^F-DCFPyL PSMA PET has a high diagnostic value for primary prostate cancer and is an effective method for the non-invasive diagnosis of prostate cancer. We hope that in the future, more prospective articles will be added to analyze whether ^18^F-DCFPyL PSMA PET can be used as a diagnostic tool to avoid prostate biopsy in patients with primary prostate cancer.

## Data availability statement

The raw data supporting the conclusions of this article will be made available by the authors, without undue reservation.

## Author contributions

WP: project development, data collection, and manuscript writing. SD: project development and data analysis. SC: data collection. ZD: manuscript writing. All authors have read and approved the final version of the manuscript.
